# Bridging language barriers in developing valid health policy research tools: insights from the translation and validation process of the SHEMESH questionnaire

**DOI:** 10.1186/s13584-023-00583-8

**Published:** 2023-11-26

**Authors:** Ligat Shalev, Christian D. Helfrich, Moriah Ellen, Keren Avirame, Renana Eitan, Adam J. Rose

**Affiliations:** 1 Braun School of Public Health and Community Medicine, Hebrew University of Jerusalem, Ein Kerem Campus, 91120 Jerusalem, Israel; 2 Seattle-Denver Center of Innovation for Veteran-Centered and Value-Driven Care, VA Puget Sound Health Care System, 1660 S. Columbian Way, S-152, Seattle, WA 98108 USA; 3 Department of Health Policy and Management, Guilford Glazer Faculty of Business and Management and Faculty of Health Sciences, Ben-Gurion University of the Negev, P.O.B. 653, 84105 Beer-Sheva, Israel; 4 Psychiatric Division, Sourasky Medical Center, 14 Weizmann Street, Tel Aviv-Yafo, Israel

**Keywords:** Validation process, Implementation science, Emergency department, Psychometrics, Organizational innovation

## Abstract

**Background:**

The use of research tools developed and validated in one cultural and linguistic context to another often faces challenges. One major challenge is poor performance of the tool in the new context. This potentially impact the legitimacy of health policy research conducted with informal adaptations of existing tools which have not been subjected to formal validation. Best practices exist to guide researchers in adapting and validating research tools effectively. We present here, as an extended example, our validation of the SHEMESH questionnaire ('Organizational Readiness to Change Assessment'; In Hebrew: 'SHE'elon Muchanut Ergunit le'SHinuy'), a Hebrew-language version of the Organizational Readiness to Change Assessment (ORCA). SHEMESH is tailored to support implementation science projects, whose aim is to promote a more rapid and complete adoption of evidence-based health policies and practices.

**Methods:**

The SHEMESH included originally eleven questions from the Evidence (item 1–4) and Context (items 5–11) domains. We validated SHEMESH through the following steps: 1. Professional translation to Hebrew and discussion of the translation by multidisciplinary committee; 2. Back-translation into English by a different translator to detect discrepancies; 3. Eleven cognitive interviews with psychiatric emergency department physicians and nurses; and 4. Pilot testing and psychometric analyses, including Cronbach’s alpha for subscales and factor analyses.

**Results:**

Following translation and cognitive interviews, SHEMESH was administered to 222 psychiatrists and nurses. Pearson correlation showed significant and strong correlations of items 1–4 to the Evidence construct and items 6–11 to the Context construct. Item 5 did not correlate with the other items, and therefore was removed from the other psychometric procedures and eventually from the SHEMESH. Factor analysis with the remaining 10 items yielded two factors, which together explained a total of 69.7% of variance. Cronbach's Alpha scores for the two subscales were high (Evidence, 0.887, and Context, 0.852).

**Conclusions:**

This multi-step validation process of the SHEMESH questionnaire may serve as a comprehensive guideline for others who are willing to adapt research tools that were developed in other languages. Practically, SHEMESH has been validated for use in implementation science research projects in Israel.

**Supplementary Information:**

The online version contains supplementary material available at 10.1186/s13584-023-00583-8.

## Introduction

### The lack of validated Hebrew-language research tools for innovations in the clinical setting

In many countries where English is not the national language, validated research tools require translation. However, the translated versions usually have not undergone a validation process, which may threaten the validity of information collected using this tool [1]. In Israel, for instance, there is a general lack of validated research tools in Hebrew for examining innovations in clinical settings, and the existing ones have not undergone a complete validation process.

For example, Tal et al. (2019) examined hospital staff members’ perceptions of adopting technological innovations. They used a translated version of a questionnaire originally developed in Spanish. To validate the questionnaire, they employed a pre-test exam among 25 physicians using the Hebrew version [2]. Although the original questionnaire had been validated in Spanish, it is unclear whether the translated version was examined for errors and what procedures were used, what pre-testing was performed before the pilot testing, and whether cultural adaptations were required.

### The importance of a validation process

A validation process helps to ensure that a research tool is performing in a way that is both valid (measuring what it is meant to measure) and reliable (measuring the same way every time) [3]. The process of validation is particularly important for research tools that were developed in another language. In that case, there are special considerations, such as the attention to cultural differences between the original and translated versions [1, 4]. Using the translation of a research tool alone without cultural adaptations may pose the risk of distorting its original meaning [5]. The translation process should ensure that the meaning and the structure of the translated version and the original one will be alike. This is not merely a pragmatical and technical task, but also requires professional skills to adapt the research tool culturally [6]. For example, the Beliefs about Medicines Questionnaire examines perceptions about the representation of medication, e.g., the necessity of a prescribed medication, or concerns about its use. The questionnaire underwent a validation process in its original language [7]. Over the years, the questionnaire was translated into different languages, and was administered in diverse cultures. Surprisingly, Garans et al. (2014) showed that the translation had different meanings for some of the items in the Norwegian, Swedish, and Danish versions. In addition, each version was far from the original version [8], calling into question whether all the different versions can truly be called the Beliefs about Medicines Questionnaire, and whether they are all measuring the same thing the same way. This example demonstrates the importance of conducting a formal and structured validation process. This is especially important when translating research tools into different languages and cultures [7], as well as for tool translations in public health field [9]. The validation methodology includes several methods, but each method has its advantages and disadvantages.

### Validation process methods: back-translation

One well-known method for validating the translation of a research tool is back-translation. This method includes a translation of the research tool from the original language, and a back translation of that version into the original language to detect inconsistencies [10].

While back-translation is a helpful tool, it is insufficient to accomplish validation by itself. The process of back-translation itself can perpetuate or even create errors [8]. Translations should also consider cultural adaptation, which can be difficult to back-translate correctly [1]. That is, a mere translation of words, even one which is correct, does not always convey the intended meaning [11]. Lastly, it is difficult to detect failures in translation and discrepancies between different translators [12].

To increase the back-translation's accuracy, it is suggested to use several independent professional translators, and to compare the original and the translated versions by a multidisciplinary committee to resolve discrepancies [1, 13]. This requires the investigators themselves to have a strong understanding of both the original and the new language, along with cultural competence.

### Validation process methods: cognitive interviews

In addition to back-translation, cognitive interviews should be used to identify and correct errors in research tools, especially questionnaires [14]. Cognitive interviewing is conducted within a small sample size, and seeks to explore how responders understand the questions and interpret them, in order to detect items whose wording may be interpreted differentially across respondents, as opposed to meaning the same thing to everyone [15]. The two techniques for cognitive interviews are: (1) *think aloud*—a respondent-driven method which the interviewees are asked to share their thoughts on their answers; and/or (2) *probing—*an interviewer-driven method which the interviewees are asked specific questions of their answers [16]. Cognitive interviews have clear benefits; however, this approach has been criticized for potential biases due to the small sample, the artificial conditions the interviews are being held, and the lack of a conceptual framework to guide the exploration and therefore the possibility for interviewer's subjectivity [17].

### Psychometric validation

The final step of the validation process is pilot testing and psychometric validation. Psychometric validation is the process of examining the statistical properties of a research tool when it is subjected to pilot testing [18], and consists of several parts. The most common maneuver is to compute coefficient alpha for the subscales of the instrument. One wants the alpha to be high, such as 0.8, which indicates a high degree of internal reliability among the items. It is also possible to give the test to the same people on different days to compute test–retest reliability, but this is not always necessary. Reporting psychometric indices such as coefficient alpha, and sometimes test–retest reliability, is an important part of being able to claim that the new instrument has been 'validated' for use. In addition, factor analyses are often used as part of this validation step [19].

In this paper we will present a case report of the translation and validation process of the SHEMESH questionnaire ('Organizational Readiness to Change Assessment'; In Hebrew: 'SHE'elon Muchanut Ergunit le'SHinuy'). SHEMESH is an implementation science research tool adapted from the Organizational Readiness to Change Assessment (ORCA), originally in English. Both the ORCA and the SHEMESH are intended to measure how favorable the environment is, at a particular study site, to introduce an innovation in care. In this instance, we plan to use the SHEMESH as part of our study of a change in practice in the psychiatric emergency department, here in Israel.

### Implementation science

Over the past three decades, it has been increasingly recognized that it is not enough to develop new treatments or prove their effectiveness [20]. There is a necessary additional step, namely to help ensure that proven treatments are adopted and sustained [21, 22]. This has led to developing a new field of inquiry called Implementation Science. The purpose of Implementation Science is to develop reproducible ways of facilitating the uniform adoption of proven clinical practices, and of addressing the many barriers that can prevent such adoption [21, 23]. Implementation Science is dedicated to better understanding the complexity of adapting interventions in healthcare settings into practice [24]. The number of investigators, publications, and grants in this field has increased many-fold over the years [24, 25], reflecting an increasing interest in it and appreciation of its importance.

### PARHIS (promoting action on research implementation in health services): a conceptual model for implementation science

Implementation science theoretical frameworks help simplify the complexity of implementation, in order to focus on key factors to measure and assess their influence [26]. One widely used framework is the Promoting Action on Research Implementation in Health Services (PARIHS) framework [27]. The PARIHS has undergone revisions as a result of empirical and theoretical work [27], but the original PARIHS framework proposed that Successful Implementation (SI) is a function of three inputs: (1) *Evidence*—end users’ assessments of the evidence strength for the innovation, including their expectations that it will be feasible to use in their setting, and applicable to their patients, and their patients’ unique needs; (2) *Context*—factors in the environment that support (or resist) the implementation of changes in practice; and (3) *Facilitation*—efforts of the research team or champions within the clinical team to promote the change. SI is the extent to which the innovation is completely implemented and adopted as part of standard practice, as opposed to incompletely adopted, or resisted [28].

### The need for a validated research tool: ORCA

The Organizational Readiness for Change Assessment (ORCA) questionnaire was developed to assess PARIHS constructs in the context of implementation studies and programs [29]. It was developed in the context of the Veterans Health Administration in the United States, but since then has been used in different settings and contexts [30–32]. The purpose of ORCA is to help apply the PARIHS framework by providing actionable measures of the key components of the framework. ORCA is based on the three PARIHS constructs, and organized in three sections: Evidence, Context, and Facilitation. ORCA has been validated for use, and its three scales had a high Cronbach's alpha reliability when subjected to validation (0.74, 0.75, and 0.95, respectively, in the original validation study) [29]. While the ORCA has been validated in English, there is currently no Hebrew version of ORCA. In fact, to our knowledge, there are no validated instruments in Hebrew for use as part of implementation science projects.

It should be noted that when one uses the ORCA in any language, the questions must be adapted anew for each new study. That is, the questions’ wording must be changed to reflect the study's setting and the innovation being adopted. Thus, no two uses of the ORCA are entirely the same. Nevertheless, much of the language in the ORCA is conserved from use to use, and therefore, one can say that the underlying ORCA has been validated [29].

ORCA is intended to support implementation projects by assessing factors related to organizational readiness to change. Measuring ORCA at baseline may help identify which sites will have difficult achieving SI; or which factors pose challenges at a given site, such as sites where there are weak perceptions of the evidence in favor of the change, or where some aspect of the underlying context is weak. These trouble spots can then potentially be addressed through facilitation. Used over time during the intervention, the ORCA can help reveal whether implementation strategies have helped improve the Evidence or Context constructs, and to what extent improvements differ among study sites [29]. A Facilitation scale can be added to ORCA at the middle or the end of a project, but this is not appropriate at baseline, before facilitation has begun [29].

### Goals of this paper

This case report study outlines the translation and validation process of the SHEMESH questionnaire. The SHEMESH will be used in our larger study examining the use of remote video-link for patient triage and admission decisions at psychiatric hospitals. The broader study is organized using the PARIHS (Promoting Action on Research Implementation in Health Services) model, and so we will be using the ORCA (Organizational Readiness for Change Assessment) as part of this project [33]. As mentioned earlier, to the best of our knowledge, there is currently no validated research tool in Hebrew that examines attitudes towards innovations in clinical settings, nor is there a research tool to collect data for implementation studies. Therefore, before using ORCA, we set out to create a Hebrew version and validate our version. We named the ORCA's Hebrew version SHEMESH, which means 'sun' in Hebrew and is the acronym derived from 'SHE'elon Muchanut Ergunit le'SHinuy', translating to Questionnaire of Organizational Readiness to Change. Once validated, the SHEMESH would be fit for use not only in our study, but as part of other Implementation Science studies in Israel. Our process, as described here, can also be an example for other Israeli researchers for how they can develop valid tools for their own research, as opposed to relying on informal and unvalidated adaptations that may compromise the validity of their research conclusions.

## Methods

### Translation process

In total, two rounds of professional translation and back-translations were conducted by two professional translators. The first translator translates from English to Hebrew, and the second from Hebrew to English. Each of them has many years of experience translating documents to be submitted to embassies, government ministries, and other similar entities. Both also have previously translated material to be used for research, including questionnaires. The first professional translator, not otherwise involved in the research, translated the ORCA from English to Hebrew. The translation was then reviewed by a multidisciplinary committee of seven members from different professional backgrounds, including implementation science and health services research (LS, CDH, ME, KA, and AJR), psychometric analysis (LS, CDH, AJR), and clinical medicine (RE and AJR). The final version was back-translated into English by a different professional translator with the expertise in psychiatric research, and reviewed by the committee for discrepancies between the original and the final version. All committee members besides one have excellent Hebrew and English. Discussion in small groups was held regarding minor wording changes (LS and AJR), psychiatry context (LS, KA, RE, and AJR), and public health relevance (LS, ME, and AJR). The overall questionnaire and any non-trivial changes were discussed among the entire research group, until agreement was reached. The full questionnaire was accepted by the committee members, and no specific concerns or comments were left for any of the questionnaire items.

Because the present manuscript is written in English, the questions presented here are a translation from the final Hebrew version of the SHEMESH by the study team (see Table 2 and Additional file 1 for the final English and Hebrew versions of the SHEMESH questionnaire).

### Cognitive interviewing

Eleven cognitive interviews were conducted by the researcher (LS) with nine psychiatrists and two psychiatric nurses who are fluent Hebrew speakers. In addition to fluent Hebrew, one of the interviewees also spoke native Russian, one native Arabic and one native Spanish. The interviews were conducted to assess the participants’ understanding of the questionnaire. Two cognitive interviewing approaches were used in relatively equal measure: *think aloud*, or asking responders to share their thoughts of the questionnaire items; and *probing,* or asking responders to paraphrase or interpret a questionnaire item. Participants were not asked to complete the questionnaire in advance, and rated the degree to which they understood the statement on a Likert scale from 1 = not understood to 5 = very much understood. The text of the instrument was changed four times in total (i.e., following every 2–3 interviews), according to the feedback received, based on discussions among the investigators as presented in the previous section, and then the new version was used for the following interview. Most of the comments received by the participants were focused on wording or styling. None of the comments emphasized concerns regarding problematic or invalid items. The cognitive interview phase ended when interviews began to produce only minor or no new suggestions.

### Pre-test sampling

The last step in the validation process was to prepare an online version of the SHEMESH, and to administer it to a sample of psychiatrists and psychiatric nurses. The sample was chosen from psychiatric hospitals in Israel. Participants at all organizational levels (administrators, physicians, and nurses) were asked to respond, since the SHEMESH is intended for all those levels. Participants were told that their participation was voluntary and that they may drop out at any stage. The study was approved by the Research Ethics Committee of Tel Aviv Medical Center.

### The questionnaire

ORCA has three constructs—Evidence, Context, and Facilitation [29]. For the purpose of the current study, because we plan to administer the ORCA before the facilitation intervention has begun, we included only the Evidence and Context constructs of the ORCA. In addition to Evidence and Context questions, we included seven questions about the responder's background (e.g., medical or nursing staff affiliation, role in the ED, number of years working at the ED). Participants were asked to rate their agreement from 1 (strongly disagree) to 5 (strongly agree) with 11 statements: (1) Evidence- four statements about the strength of the evidence, feasibility of implementing psychiatric assessment via video-link at the ED, and how preferable it is compared to the face-to-face method; and (2) Context- seven statements about the acceptability of quality improvement initiatives in one’s department or unit, the way decisions are made, and the way medical and nursing teams communicate and collaborate in the ED.

### Data analysis

Data were analyzed using SPSS (version 27.0). Descriptive statistics were used to describe the characteristics of the participants. Analysis for correlations between items was examined through Pearson correlation. Confirmatory factor analysis of the eleven items was performed using oblique rotation (Direct Oblimin). We retained factors with an Eigenvalue of 1 or greater. We also calculated Cronbach’s alpha for the Evidence and Context constructs to assess scale reliability. We used a threshold for Cronbach’s alpha of 0.7 or higher to indicate an acceptable level of reliability.

## Results

### Demographics of responders

Table 1 summarizes the demographic characteristics of the respondents. A total of 222 psychiatric and nursing staff members in fourteen psychiatric hospitals responded. Of them, 72% were psychiatrists and 28% were nurses. 47% of the responders had a management position in the hospital, although some of these people also delivered direct clinical care. 152 (72%) of the responders worked in the Emergency Department, with an average of eleven years of experience.

**Table 1:** Respondent demographic characteristics (n = 222)

	All responders (%)
Professional affiliation	
Psychiatrist	157 (72%)
Nurse	61 (28%)
Role at the hospital	
Management	102 (47%)
Team member	113 (53%)
ED worker	
Yes	152 (72%)
No. of years in ED, mean (SD)	Mean = 11.08 SD = 9.91 Median = 7.50

### Psychometric procedure

#### SHEMESH scores

Each item on the SHEMESH is on a Likert scale between 1 (strongly disagree) and 5 (strongly agree). The means, standard deviations, minimum, and maximum scores of the 11 items are presented in Table 2. The mean scores that were found ranged from 3.42 to 4.32.

**Table 2:** Score calculations of items used for SHEMESH (n = 222; min = 1, max = 5), and rotated factor analysis for the two factors according to each item of SHEMESH

Item	Mean	SD	Min	Max	Factor 1	Factor 2
1. Experts in your workplace agree with assessments via video-link	4.00	1.10	1	5	*0.924*	0.000
2. Psychiatric evaluation via video-link will be feasible	3.79	1.06	1	5	*0.923*	0.000
3. Psychiatric evaluation via video-link in the ED will be completed successfully	3.67	1.06	1	5	*0.909*	0.000
4. There will be more advantages than disadvantages for patients from conducting psychiatric evaluation via video-link	3.42	1.21	1	5	*0.852*	0.144
5. Senior staff in the psychiatric ED encourage innovation and creativity with a goal of improving patient care	4.32	0.77	3	5	−	−
6. Senior staff in the psychiatric ED encourages other members of the medical and nursing staff to share their opinions about how best to deliver care	4.07	0.93	1	5	0.000	*0.854*
7. Senior staff in the psychiatric ED encourages improved approaches to delivering clinical care	4.05	0.80	1	5	0.170	*0.820*
8. Senior staff in the psychiatric ED encourages teamwork between the medical and nursing staff to find solutions to improve patient care	4.11	0.88	1	5	− 0.149	*0.768*
9. Senior staff in the psychiatric ED encourages open communication between the medical and nursing staff	4.21	0.80	1	5	0.234	*0.717*
10. Medical and nursing staff in the psychiatric ED encourage cooperation to optimize patient care	4.22	0.74	1	5	0.196	*0.529*
11. Medical and nursing staff in the psychiatric ED have enough time to perform their work, and also to engage in quality improvement projects	3.42	1.08	1	5	0.000	*0.512*
Percent variance explained by factor:					37.7%	*27.2%*

Loading of 0.5 and higher are shaded; Item 5 has been removed from this analysis

#### Pearson correlation

We examined a Pearson correlation among the 11 SHEMESH items. The results are presented in Table 3 and indicated a strong correlation among items 1–4, representing the Evidence construct; correlations ranged from 0.562 to 0.789 (moderate to very strong). Similarly, a strong correlation was found among items 6–10, representing the Context construct, ranging from 0.403 to 0.620 (moderate to strong). Item 11, which also belongs to the Context construct, had a small degree of correlation with items 7 and 8 (0.252 and 0.220, respectively) and a stronger correlation with items 6, 9, and 10 (0.436, 0.333, and 0.521, respectively).

**Table 3:** Pearson correlations among the SHEMESH scores

Item	1	2	3	4	5	6	7	8	9	10	11
1	1	.575***	.622***	.562***	*.171*	.186*	.114	.015	.104	.176*	− .009
2	.575***	1	.789***	.724***	*.202*	.183*	.088	.046	.070	.201*	.037
3	.622***	.789***	1	.723***	*.286*	.214*	.171*	.128	.119	.249**	.064
4	.562***	.724***	.723***	1	*.319*	.259**	.197*	.071	.095	.214*	.100
5	*.171*	*.202*	*.286*	*.319*	1	*.292*	.384*	.361*	*.190*	*.221*	*.092*
6	.186*	.183*	.214*	.259**	*.292*	1	.620***	.484***	.496***	.445***	.436***
7	.114	.088	.171*	.197*	.384*	.620***	1	.646***	.570***	.434***	.252*
8	.015	.046	.128	.071	.361*	.484***	.646***	1	.742***	.403***	*.220*
9	.104	.070	.119	.095	*.190*	.496***	.570***	.742***	1	.448***	.333**
10	.176*	.201*	.249**	.214*	*.221*	.445***	.434***	.403***	.448***	1	.521***
11	− .009	.037	.064	.100	*.092*	.436***	.252*	*.220*	.333**	.521***	1

Correlations that lack a strong and/or significant correlation are in italic

*p < 0.05

**p < 0.005

***p < 0.001

These findings support our expectation that items 1–4 constitute the Evidence domain, and items 6–11 the Context domain, although item 11 had a relatively weak correlation with the other Context items compared to the other items in this scale. We further examined the effect of removing item 5 in the factor analysis as will be discussed below.

#### Factor analysis

**Fig. 1:**
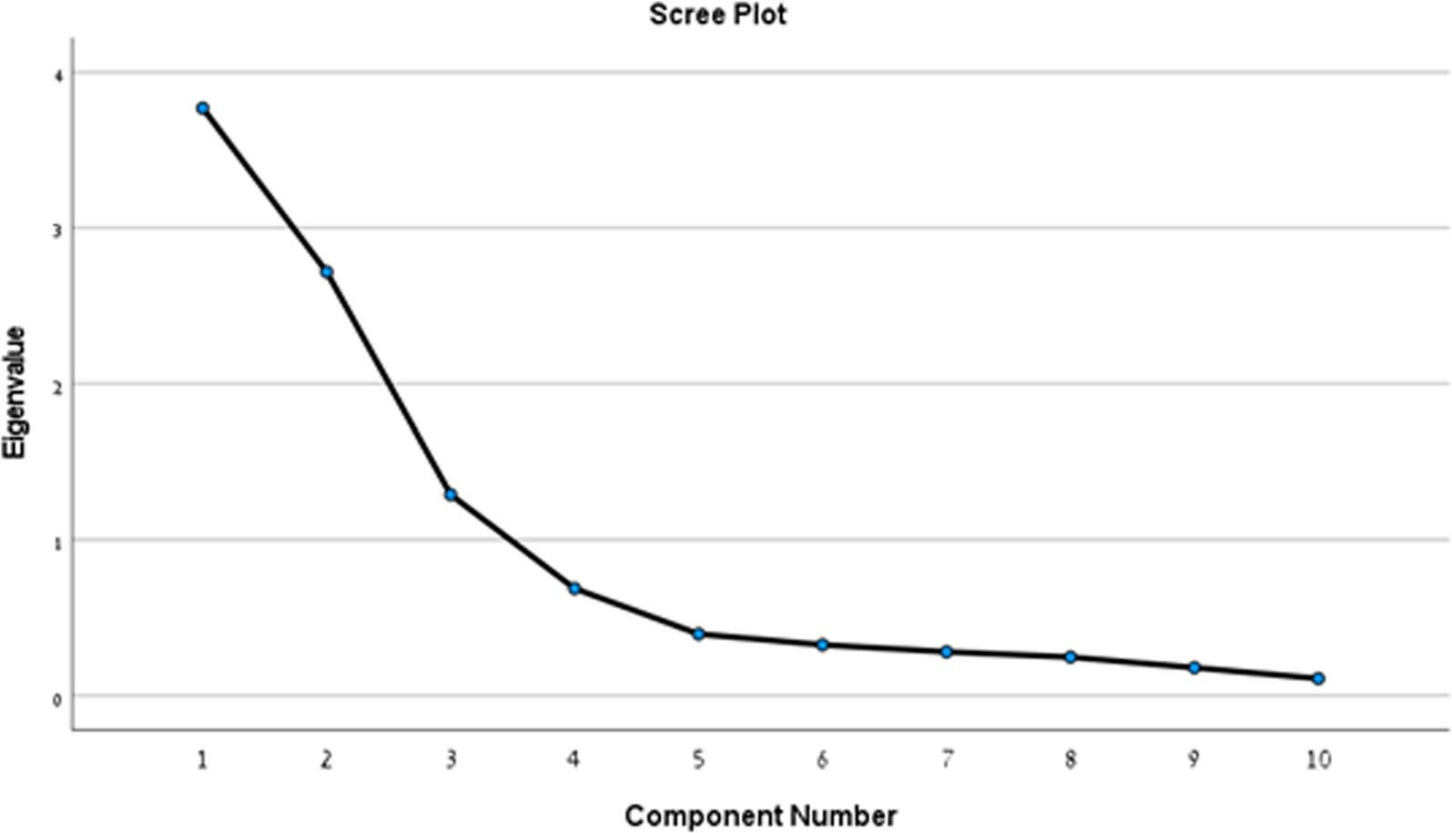
Scree plot and variance explained by 10 items in the SHEMESH (Item 5 has been removed from this analysis)

A confirmatory factor analysis of the remaining 10 items (after the removal of item 5) yielded two factors with initial Eigenvalues scores greater than one. The scree plot presented in Fig. 1 shows a result of two factors as well. The convention is to select a cutoff for factors based on the inflection point in the scree plot, based on the assumption that there will often be a discernible inflection at the point we shift between key factors that explain a lot of variance and minor factors that are catching residual amounts of variance. The first factor explains 37.7% of the variance, and the second factor the 27.2%. Together, the two factors explain a total of 64.9% of the variance. As presented in Table 2, items 1–4 loaded strongly on the first factor, and items 6–11 on the second. This again supports the idea that there are two factors, namely Evidence and Context.

#### Internal reliability

We measured internal reliabilities of the two domains of the SHEMESH: Evidence and Context. The scores for Cronbach's Alpha were high: Evidence (for items 1–4), 0.887, and Context, 0. 852 (for items 6–11, without item 5). Removing item 11 from the Context construct did not meaningfully affect the score for the Context scale (0.870).

## Discussion

The current paper describes step-by-step the translation and validation process of a research tool, and presented a case report of the SHEMESH questionnaire- the Hebrew version of ORCA, an implementation science research tool used for measuring organizational change and innovations in healthcare settings. Our study can serve as a valuable example for research teams that wish to similarly adapt an existing tool into their native language and culture. Hebrew is a language that has approximately 10 million speakers. Many potential research participants in Israel would not be able to respond to a questionnaire in English. Others might try to respond based on their limited understanding in English, but they might understand the items imperfectly. Thus, in order to have a valid tool for research, it was necessary for us to adapt an existing tool to our context. The validation process that we followed included several steps: translation and back-translation of the questionnaire, cognitive interviews, multidisciplinary expert team discussions, and pilot testing with psychometric analyses. Researchers in Israel, and in other places where English is not their first language, are urged to use a similarly rigorous process to ensure that they are using valid instruments as a basis for their research. If they base their efforts on informal and unvalidated adaptations, this may impact the validity of their research findings.

The final version of SHEMESH was acceptable to the interviewees based on their informal feedback. Our psychometric analyses indicated that all items except one did belong in the final instrument, and that there were two factors. We have removed that one item. Cronbach's alpha was high for the two final scales: Evidence (α = 0.887) and Context (α = 0.852).

Our analysis showed that the original item 5 did not correlate strongly and/or significantly to the other items in the Context scale. The item asks about the senior staff's encouragement of innovation and creativity to improve patient care. Item 7 covers extremely similar material, except it asks whether senior staff encourage improved approaches to delivering patient care, without mentioning innovation and creativity. One explanation for the finding that Item 5 did not work well may be related to the uniqueness of the psychiatric ED setting. While studies have certainly documented the importance of encouragement from management for innovations to improve care [34], actual creativity or innovation may be misplaced when undertaken by individual staff members. This may be especially true in a setting like the psychiatric ED, where medical and nursing teams must follow strict protocols in each action for the safety of the patients and themselves. That may explain why the item correlated poorly with other items we expected would favor implementation of telepsychiatry in psychiatric hospitals.

Another possible explanation for our findings with Item 5 could relate to culture. In comparison with Israel, respondents from the United States, where the instrument was developed, might have a different presumption that innovation or creativity are associated with improving quality or achieving success. Translating research tools to a different language may be adequate grammatically, but due to culture differences it may be received and understood differently [1, 4, 12]. In any event, this question was removed, and the performance of the instrument improved. This example may be instructive for research teams wishing to adapt other instruments into their native languages—there may be items that, despite the team’s best efforts, simply do not “work” well in the new form. It may be necessary to delete such items, especially if their content is already somewhat covered by other items—as was the case here.

Additionally, item 11 correlated significantly with all items in the Context construct besides items 7 and 8. Scores for items 7 and 8 were high (Mean = 4.05, 4.11; respectively); these items ask whether the senior staff encourages teamwork and improves approaches in the ED to improve patients care. On the other hand, item 11 had a moderate average score (Mean = 3.42). Item 11 asks if the psychiatric clinical staff have the time to complete their tasks as well as to engage in quality improvement projects. Looking at those items, it is understandable why the correlation may be low, since the senior staff's encouragement to improve patient care methods does not necessarily indicate that, in practice, the staff has the time to work on that. Additionally, item 11 is styled in a general manner and does not discuss specific innovation projects; therefore, responders do not necessarily have a strong opinion on that topic. Item 11 was included in the final version of SHEMESH due to its correlation with the other items in the Context domain.

Validating the SHEMESH was important, since there is a lack of validated questionnaires in Hebrew in general, and particularly to support Implementation Science projects in healthcare settings. SHEMESH is now ready for use not only in our study, but also in other implementation efforts in Israel. Those who would use SHEMESH should remember that the wording should be updated for each additional use, to reflect the setting and context of the study. For example, while our study is being conducted in a psychiatric ED, a different study might be conducted in rehabilitation facilities. While our evidence statement revolves around the use of video-link to support decisions regarding involuntary admission to psychiatry, a different study might revolve around a new method for casting fractures.

The present study has several limitations. Our sample size was limited; some studies accomplish validation of questionnaires with a larger sample size. However, our sample was sufficient to conduct our analyses and run the models we ran, and some studies have also had similar sample sizes [35]. In addition, our sample contained respondents from several hospitals, including nurses, doctors, and administrators. This variety of respondents suggests that our sample was representative and sufficient for the purpose of instrument validation. We will continue to conduct further validation analyses as we collect SHEMESH in our forthcoming study. However, we felt the need to complete and publish about a validation process before using SHEMESH for our study. An additional limitation of our study is that the Facilitation domain of ORCA was not translated and validated. We did not include this domain in our study as it was beyond the scope of our research project, since we have not yet begun our facilitation effort and therefore cannot ask about it. Validating the Facilitation items of ORCA in Hebrew would be an important direction for future research.

## Conclusions

In this paper, we discuss the methodological steps required for adapting an existing research tool into a new language and cultural context. The description of our process, and the example of how we used this process, may help to guide researchers to develop better and more valid tools than if they were to just translate an existing instrument and not validate it. The validation process we described is somewhat effort-intensive, but will hopefully result in the collection of better and more valid data, which in turn will support valid findings from health policy research.

### Supplementary Information

**Additional file 1. **The final Hebrew version of the SHEMESH questionnaire.

## Data Availability

The datasets used and/or analyzed during the current study are available from the corresponding author upon reasonable request.

## Abbreviations

ED: Emergency department
ORCA: Organizational readiness to change assessment
PARIHS: Promoting action on research implementation in health services
SHEMESH: 'Organizational readiness to change assessment'; In Hebrew: 'SHE'elon Muchanut Ergunit le'SHinuy'
SI: Successful implementation
