# Supplementary material for: Bridging language barriers in developing valid health policy research tools: insights from the translation and validation process of the SHEMESH questionnaire
Source: Isr J Health Policy Res. 2023 Nov 26;12:36. doi: 10.1186/s13584-023-00583-8 (PMC10680279; doi:10.1186/s13584-023-00583-8)
Supplement: Supplementary file 1 — Additional file 1. The final Hebrew version of the SHEMESH questionnaire. [file 13584_2023_583_MOESM1_ESM.docx]

Supplementary material- The final Hebrew version of the SHEMESH

**שאלון חדשנות בפסיכיאטריה**

***שם בית החולים בו את.ה עובד.ת*:**

**לאיזה צוות קליני את.ה משתייך.ת?** רפואה / סיעוד

**מהו תפקידך בבית החולים (ניתן לענות יותר מתשובה אחת)?**

**רופאים**- מנהל.ת בית החולים / סגן.ית מנהל.ת בית החולים / מנהל.ת מחלקה / סגן.ית מנהל.ת מחלקה / מנהל.ת יחידה / מנהל.ת שירות / רופא.ה מומחה.ית / רופא.ה מתמחה: לפני שלב א', לפני שלב ב', אחרי שלב ב'

**אחיות**-מנהל.ת שירות / אח.ות אחראית / אח.ות בצוות

**(רק למתמחים) האם כיום חלק מעבודתך (לרבות, כוננויות או תורנויות) מתקיימת בחדר המיון?** לא / כן

**האם הנך מוסמך.כת לשחרר חולים מחדר המיון ללא התייעצות עם רופא.ה כונן.נית?** לא / כן

**באילו מקרים אינך נדרש.ת להתייעצות עם הרופא.ה הכונן.נית?**

**מהו מספר שנות עבודתך בחדר המיון:**

חלק א':

בחלק הבא תתואר שיטת הערכה פסיכיאטרית חדשנית והרווחים הצפויים שיתקבלו בעזרתה:

שיטת הערכה פסיכיאטרית חדשנית: הערכה פסיכיאטרית בחדר המיון לשם אשפוז כפוי תתבצע על ידי פסיכיאטרים מומחים כוננים באמצעות שיחת וידאו במקום פגישה פנים אל פנים.

רווחים צפויים: הערכה פסיכיאטרית של רופאים מומחים כוננים באמצעות שיחת וידאו:

- תקצר את זמן ההמתנה של המטופלים בחדר המיון לעומת הערכה פסיכיאטרית המתבצעת פנים אל פנים
- כתוצאה מקיצור זמן ההמתנה יופחתו אירועי אלימות בחדר המיון

בהתבסס על הרווחים הצפויים משינוי צורת הערכה הפסיכאטרית, **באיזו מידה לדעתך יסכימו מומחים במקום עבודתך להערכה פסיכיאטרית בוידאו:**

1. הסכמה נמוכה מאוד
2. הסכמה נמוכה
3. לא הסכמה ולא סירוב
4. הסכמה רבה
5. הסכמה רבה מאוד
6. לא ידוע / לא רלוונטי

אנא ציין.ני **על סמך ניסיונך האישי** במוסד בו את.ה עובד.ת, מהי מידת הסכמתך עם ההיגדים הבאים (ממידת הסכמה נמוכה מאוד ועד מידת הסכמה רבה מאוד):

| לא ידוע/  לא רלוונטי | הסכמה במידה רבה מאוד | הסכמה במידה רבה | הסכמה במידה בינונית | הסכמה במידה נמוכה | הסכמה במידה נמוכה מאוד |  |
| --- | --- | --- | --- | --- | --- | --- |
| 99 | 5 | 4 | 3 | 2 | 1 | הערכה פסיכיאטרית בוידאו היא ברת-ביצוע ואפשרית |
| 99 | 5 | 4 | 3 | 2 | 1 | להערכתי, המעבר להערכה פסיכיאטרית  בוידאו של מטופלים פסיכיאטריים  בחדר המיון תושלם בהצלחה |
| 99 | 5 | 4 | 3 | 2 | 1 | אני משער.ת שיש להערכה פסיכיאטרית בוידאו יותר יתרונות מחסרונות **עבור המטופלים** במוסד שלנו |

**חלק ב':**

אנא ציין.ני **על סמך הניסיון האישי שלך**, עד כמה את.ה מסכים.ה (ממידת הסכמה נמוכה מאוד ועד מידת הסכמה רבה מאוד) עם ההיגדים הבאים המתייחסים **לסגל הבכיר** בחדר המיון הפסיכיאטרי שבו את.ה עובד.ת- מנהל.ת המיון וסגניו (*בשאלון הפונה אל מנהלי מחלקות הניסוח יהיה: הסגל הבכיר בבית החולים שבו את.ה עובד.ת*):

| לא ידוע/  לא רלוונטי | במידה רבה מאוד | במידה רבה | במידה בינונית | במידה נמוכה | במידה נמוכה מאוד |  |
| --- | --- | --- | --- | --- | --- | --- |
| 99 | 5 | 4 | 3 | 2 | 1 | הסגל הבכיר במיון הפסיכיאטרי מאפשר  לשאר אנשיי הרפואה והסיעוד להביע  את דעתם כשמדובר בהחלטות הקשורות  לטיפול בחולה |
| 99 | 5 | 4 | 3 | 2 | 1 | הסגל הבכיר במיון הפסכיאטרי מאפשר שיפור של שיטות הטיפול בחולה |
| 99 | 5 | 4 | 3 | 2 | 1 | הסגל הבכיר במיון הפסכיאטרי מעודד עבודת צוות בין צוותי הרפואה והסיעוד כדי למצוא פתרונות שייטיבו עם המטופלים |
| 99 | 5 | 4 | 3 | 2 | 1 | הסגל הבכיר במיון הפסיכיאטרי מעודד תקשורת בין צוותי הרפואה והסיעוד |

אנא ציין.ני **על סמך הניסיון האישי שלך**, עד כמה את.ה מסכים.ה, ממידת הסכמה נמוכה מאוד ועד מידת הסכמה רבה מאוד, עם ההיגדים הבאים על צוות המיון הפסיכיאטרי שאיתו את.ה עובד.ת:

| לא ידוע/  לא רלוונטי | במידה רבה מאוד | במידה רבה | במידה בינונית | במידה נמוכה | במידה נמוכה מאוד |  |
| --- | --- | --- | --- | --- | --- | --- |
| 99 | 5 | 4 | 3 | 2 | 1 | כלל צוות הרפואה והסיעוד במיון הפסיכיאטרי  עובד בשיתוף פעולה כדי לאפשר טיפול  מיטבי בחולה ולשפרו |
| 99 | 5 | 4 | 3 | 2 | 1 | כלל צוות הרפואה והסיעוד במיון הפסיכיאטרי  מספיק לבצע את המוטל עליו, ובנוסף גם  לעבוד על שיפור תהליכי חדשנות |
